# Applications of Ultrasound-Mediated Gene Delivery in Regenerative Medicine

**DOI:** 10.3390/bioengineering9050190

**Published:** 2022-04-27

**Authors:** Zoe Krut, Dan Gazit, Zulma Gazit, Gadi Pelled

**Affiliations:** 1 Department of Surgery, Cedars-Sinai Medical Center, Los Angeles, CA 90048, USA; zoe.krut@cshs.org (Z.K.); dan.gazit@csmc.edu (D.G.); zulma.gazit@csmc.edu (Z.G.); 2 Board of Governors Regenerative Medicine Institute, Cedars-Sinai Medical Center, Los Angeles, CA 90048, USA; 3 Department of Orthopedics, Cedars-Sinai Medical Center, Los Angeles, CA 90048, USA; 4 Faculty of Dental Medicine, Hebrew University of Jerusalem, Jerusalem 91120, Israel

**Keywords:** gene therapy, microbubbles, regenerative medicine, sonoporation, tissue regeneration, ultrasound-targeted microbubble destruction (UTMD)

## Abstract

Research on the capability of non-viral gene delivery systems to induce tissue regeneration is a continued effort as the current use of viral vectors can present with significant limitations. Despite initially showing lower gene transfection and gene expression efficiencies, non-viral delivery methods continue to be optimized to match that of their viral counterparts. Ultrasound-mediated gene transfer, referred to as sonoporation, occurs by the induction of transient membrane permeabilization and has been found to significantly increase the uptake and expression of DNA in cells across many organ systems. In addition, it offers a more favorable safety profile compared to other non-viral delivery methods. Studies have shown that microbubble-enhanced sonoporation can elicit significant tissue regeneration in both ectopic and disease models, including bone and vascular tissue regeneration. Despite this, no clinical trials on the use of sonoporation for tissue regeneration have been conducted, although current clinical trials using sonoporation for other indications suggest that the method is safe for use in the clinical setting. In this review, we describe the pre-clinical studies conducted thus far on the use of sonoporation for tissue regeneration. Further, the various techniques used to increase the effectiveness and duration of sonoporation-induced gene transfer, as well as the obstacles that may be currently hindering clinical translation, are explored.

## 1. Introduction

The field of regenerative medicine was established with the goal of regrowing human tissues to restore endogenous function following traumatic injury or disease [1]. The global popularity of this interdisciplinary field has resulted in a paradigm shift from palliation to an emphasis on restorative therapies [2]. Gene delivery, the administration of genetic material to modify gene expression, presents a promising approach to accomplish this goal across various disease states. However, identifying safe delivery vectors capable of producing a sustained biological effect, crucial for clinical implementation and success, remains a major challenge to this day [3,4]. 

There are two fundamental gene delivery systems: viral and non-viral [5]. Viral vectors utilize the ability of viruses to introduce their DNA into host cells, a process called transduction. While capable of successfully yielding gene expression due to the ability of the viral structure to prevent degradation, several studies have demonstrated that the use of these carriers presents several limitations, including immunogenicity [6], off-target delivery [7], and difficult vector production [8]. Despite advances to improve the safety of viral vectors [9,10,11], the development of effective non-viral delivery systems is still needed [12].

Non-viral approaches include the injection of naked DNA, the extended-release of DNA from biodegradable scaffolds, known as gene-activated matrices (GAMs) [13,14,15], complexation with various chemical agents that facilitate membrane penetration, and the use of physical methods, including electroporation, sonoporation, and magnetofection [16]. This review focuses on sonoporation, while these other methods have been detailed elsewhere [17,18]. Table 1 provides a brief overview of the advantages and disadvantages of the non-viral gene delivery systems mentioned above.

Within the last two decades, the use of sonoporation-based gene delivery for tissue regeneration has gained traction, and while promising results have been previously demonstrated, further improvements are still being made. In this review, we will focus on the use of sonoporation for tissue regeneration and outline the in vivo studies conducted thus far.

## 2. Sonoporation-Based Gene Delivery

**Figure 1:**
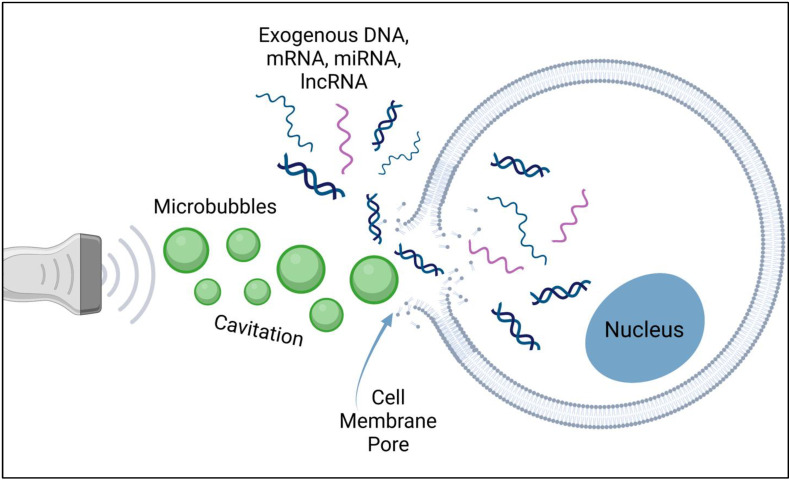
Image depicting the induction of a pore in the cell membrane by cavitation of microbubbles, allowing exogenous nucleic acids to passively diffuse into the cytoplasm. Created with BioRender (BioRender.com, accessed on 20 April 2022).

Sonoporation has been shown to be an effective non-viral gene delivery system in both in vitro and in vivo studies across many organ systems [23,24,25]. In comparison to some non-viral methods, however, sonoporation is noted as having lower gene transfer efficiency and may also cause cells to undergo apoptosis due to damage to the cell membrane [23]. The use of ultrasound energy to enhance gene delivery into targeted cells was first evaluated in the 1980s [26]. Ultrasound waves can modify the permeability of the cell plasma membrane, and this characteristic can be applied to gene delivery. In order to enhance the efficiency of sonoporation-based gene delivery, the use of ultrasound contrast agents, microbubbles, was explored and has since become quite commonly used. The ultrasonic waves induce cavitation, the growth, oscillation, and collapse of small gas bubbles in a fluid, which was first visualized in 1999 using scanning electron microscopy [27]. Cavitation results in greater permeabilization of cell membranes, allowing for nucleic acids to passively diffuse into the cytoplasm through cavitation-induced pores (Figure 1). Moreover, the use of microbubbles enables real-time monitoring of the sonoporation process on the ultrasound screen.

In addition to serving as a contrast agent in medical imaging [28], first approved by the United States Food and Drug Administration (FDA) in 1994 [29], microbubbles have been shown to enhance sonoporation. When used in conjunction with microbubbles, ultrasound-induced cavitation effects increase the efficiency of DNA uptake through cavitation-induced pores. Studies have been conducted in which microbubbles have been excluded, and plasmid DNA was injected with low-intensity pulsed ultrasound (LIPUS) alone [30], but transfection was far superior when microbubbles were incorporated [31]. In certain models, without the synergistic effect between microbubbles and ultrasound, effective gene transfer and expression did not occur at all [32].

Both in vitro and in vivo studies have shown that sonoporation outcomes are significantly impacted by the power settings on the ultrasound, the duration of ultrasound exposure, and the plasmid and microbubbles being injected [27,33,34,35]. One study found that there was an inverse relationship between microbubble concentration and cell viability [36], while another suggested that the use of microbubbles during sonoporation was capable of reducing the skeletal muscle damage observed when naked DNA is injected directly into the tissue [19]. In addition, the type of microbubbles used may impact the resulting gene expression and cell viability. Biotinylated cationic microbubbles appear to be preferable to neutral forms due to their more efficient binding of both cells and nucleic acids, and they also seem to provide protection when the ultrasound power density is increased, although only a few studies have conducted a direct comparison [32,36].

Current studies are trying to find a balance between cell viability and gene expression. In attempts to prolong expression in vivo, up to 85 days, it appears that the plasmid being used has a role to play in the duration of gene expression [37,38]. Studies have found that the delivery of the gene through an implanted scaffold or matrix may enhance sonoporation-induced gene transfection at the target site up to 25-fold [32]. A scaffold may also attract endogenous progenitor/stem cells to the injury site [39,40] or provide a protective factor for implanted stem cells, the targets of transfection [32,41]. However, further studies are warranted to determine how to overcome the possible ultrasound wave attenuation caused by the implanted materials [32,42]. 

## 3. Applications of Sonoporation for Tissue Regeneration

The use of sonoporation for tissue regeneration has been characterized in various animal models (Table 2). 

### 3.1. Sonoporation for Skeletal Tissue Regeneration

Despite the remarkable regenerative capacity of bone tissue, there are several instances where biological processes are unable to fully recuperate and repair bone loss, leading to conditions such as nonunion fractures [57]. Preliminary studies on sonoporation focused on the ultrasound parameters necessary to achieve efficient gene transfection when injecting naked plasmid DNA into skeletal muscle [48]. These parameters were adopted for use in a 2008 study by Sheyn et al., the first in vivo study on the use of sonoporation for skeletal tissue formation [22]. Published less than 10 years after it was demonstrated that sonoporation induces membrane permeabilization, this study found that the intramuscular injection of a plasmid encoding recombinant human bone morphogenic protein-9 (rhBMP-9), mixed with lipid-stabilized microbubbles, resulted in ectopic bone formation in 6 out of 8 mice treated with sonoporation. However, this study compared these results to electroporation and found that all mice that received electroporation experienced ectopic bone formation. When comparing the ectopic bone formed in each treatment group, the bone volume in electroporation-treated mice was significantly larger than the bone generated by sonoporation. Conversely, the bone volume density of the newly formed tissue generated by sonoporation was found to be significantly larger than that formed by electroporation. Even though it was found to be less efficient than electroporation, this study demonstrated that sonoporation was capable of inducing bone regeneration. The authors also indicated that sonoporation did not exhibit the same adverse effects seen with electroporation, namely muscle tissue damage. Other studies have found that while electroporation increased transfection and induced bone regeneration [58], it can be accompanied by other undesirable effects, such as passive involuntary muscle contractions, tissue damage associated with thermal changes at the site of electric pulses, and non-homogenous tissue regeneration [59,60].

A year after the initial Sheyn et al. study was published, researchers in Japan utilized a plasmid-based human BMP-2 construct and transcutaneous sonoporation in male mice [43]. Sonoporation with lipid microbubbles was repeated at 24-h intervals for up to 7 days. By 21 days after the final ultrasound treatment, cartilage and immature bone were observed in the gastrocnemius muscle, and evaluation of muscle fibers revealed bone matrix with bone marrow that included blood cells and adipocytes. In addition, alkaline phosphatase (ALP) activity increased significantly and the Ca^2+^ concentration was higher, indicating that sonoporation had indeed caused osteoinduction. This study demonstrated that repetition of this procedure significantly increased osteoinduction in the target muscle compared to only one session of sonoporation. This is consistent with other studies that have used sonoporation for gene delivery for other disease models, including cancer [61]. It is important to note, however, that repeated intramuscular injection of naked plasmid DNA can cause various degrees of muscle damage and inflammatory responses, even when not applying ultrasound, and thus, repeated injections may introduce additional risks [62].

In an attempt to increase the effectiveness of sonoporation, the use of a matrix, referred to as matrix-assisted sonoporation (MAS), was evaluated by Nomikou et al. [31]. Published in 2018, the researchers utilized an advanced ultrasound-responsive gene-activated matrix (GAM). This matrix, injected into the hind leg of mice, was composed of fibrin and collagen and contained polymeric microbubbles, pVAX-BMP2/7 co-expression plasmids, and C2C12 mouse myoblast precursor cells. Following external application of the ultrasound, enhanced ectopic bone formation was evident in 100% of mice treated with GAM and ultrasound, with a 5.7-fold increase compared to passive GAM (no ultrasound) and a 16.44-fold increase compared to standard sonoporation (injection of DNA and microbubbles followed by ultrasound, no GAM/exogenous cells). An interesting finding from this study was that only 6.5 µg of DNA were needed to elicit this response; a previous study from 2014, the first to evaluate sonoporation with BMP2/7 co-expression plasmids in a bone defect model, found it necessary to use 100 µg of DNA in multiple consecutive injections, along with a much higher ultrasound energy density [35]. In this study by Feichtinger et al., results included irregular ectopic bone structure, multiple centers of ossification, and skin burning at the exit site of the ultrasound when using a 4 W/cm^2^ protocol. A possible advantage of using a GAM is that while formulated as a liquid, the fibrin gelling time can be adjusted to occur directly after injection, and this may minimize off-target bone generation.

The study by Nomikou et al. emphasized the importance of specific “responder” cells at the target site. This point was further demonstrated by a study in Yucatan mini-pigs with a critical-sized bone fracture in the tibia, where a collagen scaffold was then implanted to facilitate the recruitment of endogenous mesenchymal stem cells (MSCs) into the fracture site [39]. Two weeks after the defect was created, transcutaneous ultrasound-mediated delivery of hBMP-6 plasmid led to complete radiographic and functional fracture healing in all animals at 6 weeks post-sonoporation. In control animals, nonunion was evident. This study, the only one that has reported on the use of sonoporation for tissue regeneration in large animals thus far, suggested that the delayed administration of BMP-6 may have allowed for sufficient endogenous progenitor migration and retention in the scaffold at the target site prior to treatment. This approach has been previously suggested in a study that delayed the administration of a BMP-2-encoding adenoviral vector and found improved bone formation in a critical-sized femoral fracture in rats [63]. Compared to the sonoporation group, similar results were found in animals treated with a bone autograft, the current standard of care, suggesting that the sonoporation method used was as efficient; the authors also noted that the bone autografts used in this experiment might be far superior to autografts typically available in the clinical setting [39]. 

Targeting endogenous progenitor/stem cells using sonoporation was also demonstrated in skeletal soft tissues. Delalande et al. had previously established optimized parameters for effective gene expression to tendons [64]. In a separate study, an anterior cruciate ligament (ACL) reconstruction procedure was conducted in Yucatan mini pigs. Allogeneic tendon grafts were secured in femoral and tibial bone tunnels, and a collagen scaffold was implanted around the grafts to attract endogenous progenitor/stem cells [40]. Two weeks after the tendon graft and scaffold implantation, transcutaneous ultrasound was used to deliver a BMP-6 gene. Results showed that, in addition to a 15-fold increase in the expression of BMP6 in animals treated with ultrasound, osteointegration of the tendon grafts was significantly enhanced. Animals treated with sonoporation exhibited twice the bone volume as the control animals, and the researchers noted tissue continuity and a lack of ectopic bone formation. The absence of mononucleated cells in the grafts post-mortem suggested that sonoporation did not invoke an inflammatory reaction.

Table 3 summarizes the studies that have used sonoporation for skeletal tissue regeneration thus far. Despite promising results, improvements in transfection efficiency are still desired. Studies have found that adjustments to ultrasound parameters can increase gene expression in the bone defect model for up to 21 days [65]. However, sonoporation is especially challenging in sites involving bone and metal implants, such as those used in the fixation of fractures, as they are highly reflective of ultrasound waves. Hence, efficient transfection may require longer ultrasound times [64], but this may lead to adverse side effects.

### 3.2. Treatment of Myocardial Ischemia with Sonoporation

Cardiac wound healing in mammals is severely limited due to the development of scar tissue and the necrosis that results due to the loss of blood supply [66]. To try to address this lack of self-regeneration, a preliminary study from 2012 used ultrasound and microbubbles to deliver the thymosin beta 4 (TB4) gene under a piggyBac transposon plasmid to normal rat hearts [67]. TB4 was found to stimulate angiogenesis and arteriogenesis and promote the proliferation and differentiation of resident WT1-positive adult cardiac progenitor cells into three intact cardiac cell lineages: vascular endothelial cells, coronary artery smooth muscle cells, and cardiac muscle cells. This study provided support for the use of sonoporation for cardiac regeneration, whereas the first study to demonstrate this was published in 2009 [44]. In this study from 2009, lipid microbubbles and plasmid DNA were injected intravenously into mice 7 days after coronary artery ligation, which is used to model a myocardial infarction (MI). The plasmid injected was encoded for either vascular endothelial growth factor (VEGF) or stem cell factor (SCF). Results from this study showed that injection of either plasmid yielded greater capillary and arteriolar density, myocardial perfusion, and enhanced cardiac function compared to the control group, which received empty plasmids. A follow-up study was conducted by the same authors to determine the effect of multiple treatments in the MI rat model [46]. They found that multiple injections of SCF and stromal cell-derived factor-1α (SDF-1α) resulted in the greatest increase in vascular densities compared to the control, although all sonoporation recipients did exhibit an increase in vascular density and a smaller infarct region. Myocardial perfusion and ventricular function also improved progressively with the number of treatments.

Soon after, the use of cationic microbubbles was compared to commercially available lipid microbubbles. This comparison was assessed based on the ability to deliver the therapeutic AKT gene, a serine/threonine-protein kinase, to ischemic rat myocardium via intravenous injection [47]. The authors found that the cationic microbubbles bound 70% more plasmid DNA, and they attributed this to the high zeta potential of cationic microbubbles. In an ischemia/reperfusion (I/R) rat model, the authors demonstrated that AKT transfection reduced infarct size, increased infarct thickness, and reduced apoptosis. It was also found that ejection fraction was significantly improved and that the densities of the capillaries and arterioles within the border region of the infarct were significantly increased compared to the control animals treated with a vector control or lipid microbubbles. The study concluded that cationic microbubble-based delivery of the AKT gene produced the greatest increase in ventricular function and myocardial perfusion.

Unlike previous studies, which injected a plasmid, a more recent study aimed to increase the delivery of antagomir to the myocardium of both healthy control mice and in an I/R injury mouse model [45]. Antagomir is a microRNA (miRNA)-inhibitor, but it has low myocardial specificity, and thus, cardiac treatment with antagomir requires high doses, which can result in adverse side effects. The researchers found that ultrasound and cationic microbubbles significantly increased local antagomir delivery to the non-ischemic heart; they also noted only modest side effects, including neutrophil invasion, but did not observe an increase in apoptosis. The findings from this study also suggest that antagomir enters cardiomyocytes within 30 min post-treatment and remains there for at least 48 h. After I/R injury, antagomir readily enters the infarcted zone, but the results did not show any additional regenerative benefits when using the ultrasound. Interestingly, this study demonstrated that the extent and location of antagomir delivery were dependent on the ultrasound frequency and mode. While delivery occurred mostly to the anterior wall of the heart, a higher frequency led to more restrictive delivery to the anterior wall, while a lower frequency enabled delivery to more parts of the heart. In support of the proposed safety of sonoporation, this study found that any damage to the heart was local and temporal and that treatment did not cause damage to the cardiomyocytes themselves.

The use of miRNA-inhibitors (antimiR) and sonoporation has also been proposed to prevent the destruction of heart tissue. Researchers found that multiple treatments with cationic microbubbles and antimiR-23a in a phenylephrine-induced cardiac hypertrophy mouse model resulted in a 41% decrease in cardiac miR-23a levels, a decrease in the mass of the left ventricle, and a higher fractional shortening [68]. Cardiac levels of hypertrophic mRNAs (ANP and MYH7) also decreased, but this difference did not reach statistical significance. This suggests that ultrasound significantly reduces the antimiR dose needed for therapeutic efficacy by a factor of over 200-fold. However, this does not address whether treatment can cause a regression in established or chronic cardiac hypertrophy. In addition, the researchers saw a loss of protection against left ventricular hypertrophy by one-week post-ultrasound treatment, with ongoing phenylephrine infusions, but did note increased protection against systolic dysfunction during ultrasound treatments.

Similar to the use of sonoporation for skeletal tissue regeneration, the optimal ultrasound parameters and injection method for cardiac transfection still need to be confirmed. For example, a study in 2015 aimed to transfect microRNA-21 (miR-21) in healthy swine hearts and wanted to compare the efficacy of intravenous injection to that of injection directly into the myocardium [69]. The authors found that an ultrasound intensity of 2 W/cm^2^ and a 50% duty ratio for 20 min was best and did not cause any injury to the myocardium. Using these parameters, they found an increase in gene expression in the myocardium, regardless of the miR-21 delivery route, but they did note a slight increase in transfection efficiency with intracoronary injection; it is important to note that this increase in transfection efficiency was observed even when the dose for the intracoronary injection was half that of the intravenous injection. This study also suggested that preconditioning-regulated miR-21 could protect the heart against injury via anti-apoptosis through its target PDCD4. 

In addition, in an effort to increase the efficiency of gene delivery for cardiac regeneration, a recent study explored the use of sonoporation for the delivery of therapeutic genes to an MI rat model, using microbubbles conjugated to adenoviruses encoding for the Ca^2+^ ATPase 2a (SERCA2a) and connexin 43 (Cx43) genes [70]. This study showed that animals that received both genes had the best cardiac contractile function and electrical stability compared to controls, although there was evidence of a change in the infarct size. Interestingly, the therapeutic efficacy was further increased when bone marrow MSCs were injected at the infarct site and border zones 4 weeks prior to sonoporation. The authors did not determine whether the injected cells were subsequently transfected during the sonoporation process, but they did hypothesize, based on previous studies, that the stem cells helped to maintain an important population of cardiomyocytes that were targeted for transfection. This study further supports the importance of targeting the right responder cells at the site that requires regeneration and proposes the potential benefit of using sonoporation in conjunction with other gene delivery systems to maximize the resulting therapeutic effects. Table 4 summarizes the studies that used sonoporation for cardiac tissue regeneration, but it is evident that additional research is needed.

### 3.3. Treatment of Peripheral Ischemia with Sonoporation

Like the biological limitations of resolving a myocardial infarction, peripheral artery disease also cannot be resolved through self-regeneration. In a rabbit peripheral ischemia model, hepatocyte growth factor (HGF) plasmid and lipid microbubbles were injected locally into the pretibial muscle, followed by ultrasound application [48]. Five weeks after transfection, the angiographic score and capillary density of animals treated with ultrasound significantly increased compared to those only injected with the HGF plasmid. This was accompanied by a significant increase in blood flow and in the blood pressure ratio. This study provided evidence that sonoporation could be a safe and useful gene-based therapy to treat peripheral artery disease. 

In a rat model of severe chronic hindlimb ischemia, a study showed that VEGF-165 plasmid, infused intravenously over 10 min, and ultrasound application resulted in a significant improvement in microvascular blood flow and in an increase in vessel density [49]. Improvement in tissue perfusion was attributed to increases in noncapillary blood volume (arteriogenesis), with perfusion peaking at 14 days post-treatment, followed by a partial regression of neovascularization at 6 weeks. Transfection was localized primarily to the vascular endothelium of arterioles. Later, a similar study design compared the efficacy of treatment with different sites of injection, intravenous (IV) and intramuscular (IM) [50]. They found that both IM and IV delivery produced significant increases in microvascular blood volume and microvascular blood flow, but microvascular blood flow was greater in IV-treated animals, even 8 weeks post-treatment. Interestingly, VEGF165/GFP mRNA expression was significantly greater in IM-treated animals. Intravenous delivery resulted in directed vascular transfection over a wider distribution, which may account for the greater degree of angiogenesis in this group. 

More recently, a study was designed to compare temporally separated VEGF and angiopoetin-1 (Ang-1) delivery to concomitant delivery and single VEGF delivery for therapeutic angiogenesis in a chronic unilateral hindlimb ischemia rat model [51]. Using cationic microbubbles, the authors found that VEGF delivery improved blood flow and vessel density, but flow reserve remained low, and supporting cell coverage in the new vessels was poor. VEGF/Ang-1 co-delivery marginally increased blood flow and vessel density, but the authors did note that co-delivery improved flow reserve and supporting cell coverage. Temporally separated VEGF and Ang-1 delivery, with VEGF delivered at 2 weeks after ligation and Ang-1 delivered at 4 weeks post-ligation, resulted in increased blood flow and vessel density and increased and sustained the flow reserve, with improved pericyte coverage at 8 weeks.

Lastly, Cao et al. published a sonoporation study in 2015, using miR-126-3p and cationic microbubbles in rats following chronic left femoral artery ligation [52]. Treatment of the chronic ischemic hindlimb muscle resulted in improved perfusion and vessel density, enhanced arteriolar formation, pericyte coverage, and phosphorylated Tie2 levels and did not affect miR-126-5p or delta-like 1 homolog levels. The authors speculated that the observed biological effect was a result of suppressing sprouty-related protein-1 (SPRED1) and phosphatidylinositol-3-kinase regulatory subunit 2 (PIK3R2) and enhancing VEGF and Ang-1 signaling. This study also showed that the use of cationic microbubbles stabilized and extended the circulatory time in vivo, finding that conjugating the microbubbles and miRNA prior to injection led to prolonged circulatory time compared to that of unbound miRNA. Overall, this study showed that treatment with miR-126-3p resulted in significant improvements in microvascular perfusion, with minimal to no uptake in remote organs. Animals that underwent repeated injection and ultrasound treatment exhibited an even greater angiogenic response. Table 5 summarizes the studies that have explored the use of sonoporation as a therapy for peripheral ischemia thus far.

### 3.4. Sonoporation for Pancreatic Islet Regeneration

Pancreatic islets contain multiple cell types, including beta (β) cells that produce insulin, and are therefore a treatment target in the effort to reverse diabetes mellitus. The first study that demonstrated the ability of ultrasound-targeted gene therapy to regenerate pancreatic islets, thus leading to recovery from diabetes, without the use of a viral vector, was published in 2010 [53]. Chen et al. utilized lipid-stabilized microbubbles with a modified rat insulin promoter (RIP3.1) for greater targeting to β-cells, in a streptozotocin (STZ)-induced diabetic rat model. Several genes were used, but while PAX4, Nkx2.2, Nkx6.1, Ngn3, and Mafa produced alpha-cell hyperplasia, there was no significant improvement in the β-cell mass or in blood glucose levels 30 days post-sonoporation. In contrast, injection of RIP3.1-NeuroD1 promoted islet regeneration from surviving β-cells, resulting in the normalization of glucose, insulin, and C-peptide levels at 30 days post-treatment. In a longer-term experiment, 4 out of 6 rats had a return of diabetes by 90 days, which was accompanied by β-cell apoptosis. However, for rats pretreated with SP600125, a JNK inhibitor, β-cell apoptosis was restricted, and researchers noted an extension in the duration of islet regeneration and normoglycemia to 90 days, suggesting that an immunosuppressive regimen for islet protection may need to be established going forward. 

A similar study from this group delivered islet transcription factor genes using a piggyBac transposon gene delivery system for long-term transgene expression of Nkx2.2 in the pancreas of an adult diabetic rat model [54]. Results showed that the Nkx2.2 gene induced robust proliferation and differentiation of adult pancreatic progenitors. Using high-resolution confocal images, the authors were able to show how one differentiated pancreatic progenitor cell developed into islet-like clusters, and then into mature islets with normal morphology. This pancreatic islet regeneration process enabled a reversal in STZ-induced diabetes for 3 months.

Previous work has shown that gene therapy with cyclin D2/CDK4/GLP-1 plasmids targeted to the pancreas of STZ-treated rats could force cell cycle re-entry of residual G_0_-phase islet cells into the G_1_/S phase in order to regenerate β cells [55]. A single treatment of sonoporation induced β-cell regeneration with reversal of diabetes for 6 months, without evidence of toxicity or the activation of oncogenes. Cyclin D2/CDK4/GLP-1 gene delivery initiated robust proliferation of the adult pancreatic progenitor cells that exist within islets. A similar study used the ANGPTL8 gene, delivered to the pancreas, liver, and skeletal muscle of normal adult rats [56]. ANGPTL8 was detected in circulation 1-month post-treatment. Sonoporation with ANGPTL8 significantly alleviated but did not totally reverse STZ-induced diabetes in this rat model. The largest improvement, however, was seen when ANGPTL8 was targeted to the pancreas. Pancreas-targeted treatment promoted the proliferation of adult and aged β cells, expanded the β cell mass, improved glucose tolerance, and increased the fasting blood insulin levels without causing severe hypertriacylglycerolaemia. The authors suggested that the lack of disease reversal may be due to the fact that the treated animals did not have enough β cells to undergo replication in sufficient numbers to actually reverse the disease. 

A more recent study hypothesized that gene therapy with a plasmid cDNA cocktail of BMP7/PRDM16/PPARGC1A, injected into the skeletal muscle of obese Zucker diabetic fatty rats, would produce a brown adipose tissue phenotype with UCP-1 overexpression [71]. This proof-of-concept project used lipid-stabilized microbubbles injected intravenously. Treatment produced a reliable pattern of enhancing skeletal muscle overexpression of the UCP-1 gene, indicating that this plasmid DNA construct may be capable of reprogramming adult skeletal muscle tissue into brown adipose cells in vivo. Table 6 provides an overview of the studies that have demonstrated the ability of sonoporation to induce the regeneration of pancreatic islet cells, but this study highlights the additional ways in which sonoporation may be used to ameliorate the diabetic condition, supporting the need for additional research in this area. 

### 3.5. Other Applications of Sonoporation for Tissue Regeneration

In addition to the studies detailed above, sonoporation has also been tested in other models of tissue regeneration. The use of sonoporation for dental conditions is desirable because current gene delivery methods exhibit low transference and efficiency in the pulp space. In 2002, Nakashima et al. aimed to use gene therapy to induce reparative dentin formation for potential pulp capping since the conventional method, using calcium hydroxide, only induces a small amount of reparative dentin [72]. This group of researchers had previously optimized the transference of a growth/differentiation factor 11 (Gdf11)/BMP11 plasmid into dental pulp stem cells by in vivo electroporation but also noted an accumulation of erythrocytes in a plasma clot adjacent to the electrode, potentially caused by thermal effects. As a result of these findings, the authors hoped to eliminate the adverse effects by utilizing sonoporation instead [60]. While therapeutic reparative dentin formation can be achieved with BMPs [73], the half-life of the morphogen is a limiting factor, and the BMPs must reach the pulp tissue stem cells in order to be effective. Sonoporation of Gdf11 stimulated a large amount of reparative dentin formation in the amputated dental pulp of canine teeth in vivo. This was a promising result, suggesting that sonoporation may be useful in endodontic dental treatment, although no in vivo dental regeneration studies have been conducted since. In 2014, Sugano et al. were able to optimize the intensity and exposure time of sonoporation for treating periodontitis in rats, although this study was not designed to demonstrate tissue regeneration, and thus, did not use genes relevant for regeneration [74].

Two recent studies have investigated how the principles of sonoporation can be used to increase hair follicle growth [75,76]. Liao et al. focused on the potential of inhibiting bacteria and allergies on the scalp with the use of lysozyme-shelled microbubbles, finding that ultrasound application significantly enhanced hair growth rates in mice [75]. Ryu et al. designed a microbubble-nanoliposomal particle to act as a Cas9/sgRNA riboprotein complex carrier and found that the protein constructs were successfully transferred into dermal papilla cells, effectively treating the mice with androgenic alopecia [76]. The results of this study demonstrated that the external application of ultrasound allowed the treatment to diffuse deeper and that the riboprotein complex carrier experienced a high efficiency of recognition and gene editing, with an on-target effect of about 70%. The authors of this study encouraged future research on how this method could be applied to the treatment of skin carcinoma and melanoma.

Sonoporation has also been used to reverse the innate reparative processes of the body, such as the creation of fibrotic tissue. Cirrhosis, a chronic liver disease, occurs due to the progressive deterioration of liver function and results in fibrosis, which may lead to liver failure. A study found that intravenous delivery of the hepatocyte growth factor (HGF) gene and application of ultrasound markedly attenuated fibrosis in rats and resulted in a significant decrease in the serum levels of alanine transaminase (ALT) and aspartate transaminase (AST), and the expression of collagen I, collagen II, and α-SMA proteins [77]. Soon after this study was published, another examined the antifibrotic effect of an artificial microRNA designed to target a connective tissue growth factor (CTGF). Conjugated with cationic microbubbles, using a biotin-avidin system, this study showed that repeated delivery of artificial miRNAs is safe and convenient, and the results demonstrated that the progression of hepatic fibrosis was effectively ameliorated, and cirrhosis was successfully prevented by downregulating CTGF expression [78]. Despite effective delivery to the liver, however, liver injury was not reversed. This finding suggests that the induction of healthy tissue growth may not always be sufficient to reverse a chronic condition and that efforts to address innate scarring processes, for example, may also need to be employed.

## 4. Considerations for Clinical Translation

Currently, sonoporation is only cited in three ongoing clinical trials. Although up to 115 studies reference the use of ultrasound and microbubbles, most studies focus on the imaging and diagnostic applications or are using ultrasound-induced cavitation for thrombolysis. However, of the three ongoing clinical trials using sonoporation, none utilize sonoporation for gene delivery nor for tissue regeneration. The current clinical utilization of sonoporation is to enhance the targeted delivery of chemotherapeutic agents [79]. The preliminary results from these trials, including one in which sonoporation is used to disrupt the blood–brain barrier in patients with recurrent glioblastoma [80], demonstrate that systemic delivery of microbubbles and repeated ultrasound exposure is a safe practice for humans.

That said, there are various factors which need to be addressed further before this application of sonoporation can be translated to the clinical setting. These factors primarily pertain to the specifics of the microbubble and plasmid injection. The current body of research has not been able to conclusively identify the best type of microbubbles to use, although it appears that cationic microbubbles may increase transfection compared to lipid-coated microbubbles [32,47], nor have researchers been able to establish the best timeline for gene delivery [51]. While it appears that there are benefits to the co-delivery of genes [31,35,81], it will be important to determine the best ratio of the genes and whether, indeed, the genes should be delivered at the same time or if they should be delivered at separate times [51]. In addition, especially in cases when a scaffold is utilized in the target site, while it has been found that delayed administration of plasmid DNA allows for sufficient endogenous progenitor cell migration [39,40,63,64], it will be necessary to determine the most optimal delay in sonoporation.

Clarification on when the ultrasound should be applied, with respect to the injection, is also needed. Some studies have utilized continuous infusion during ultrasound application [47], while others have completely infused the plasmid and microbubbles and then applied the ultrasound [44]. It may be the case that the ultrasound should be applied during infusion and then for a set time after the infusion has been completed to ensure that all microbubbles still in circulation have been destroyed [49]. In addition, while repeated treatments have been found to be safe and may result in greater regeneration [43,46], the timeline of treatment must be assessed further, as there is still the risk of muscle damage and inflammatory reaction with each intramuscular injection [62].

Additionally, the site of injection is important to study further; while the majority of studies utilize intravenous injection, it will be important to deduce how direct injection impacts transfection efficiency, as one study found that intracoronary injection of a half-dose resulted in slightly higher transfection efficiency than intravenous injection [69]. Despite this, intravenous injection appears to be a safe practice and may present the least burdensome injection method, which would further support the adoption of this method in the clinical setting.

The location of where the ultrasound is applied may also need to be studied further. Transcutaneous sonoporation has been found to successfully induce osteoinduction [43] but may be limited in its clinical use for fractures located in deeper tissues, such as in the case of hip fractures [82]. The concept of implantable sonoporation devices has not yet been heavily studied for tissue regeneration, but cases that have used sonoporation for blood–brain barrier disruption in the treatment of brain disorders have found it to be beneficial. Particularly, implantation enables researchers to avoid the large ultrasound wave attenuation caused by the skull when ultrasound is applied externally [80].

While sonoporation has been found to have minimal to no off-target delivery, studies have demonstrated that the frequency and mode of the ultrasound may alter the extent of delivery to certain tissues. This was seen in the study by Kwekkeboom et al., where delivery primarily occurred to the anterior wall of the heart unless a lower frequency was used [45]. Despite the current collection of pre-clinical studies on the induction of tissue regeneration by sonoporation, there is a clear need for greater guidance on how to optimize the therapeutic benefits, although this may likely differ depending on the targeted organ system. 

## 5. Conclusions

The application of ultrasound as a method to increase gene delivery is advantageous because ultrasound has already been integrated into clinical practices, and it allows for the visual monitoring of the transfection process when microbubbles are co-injected. While the in vivo studies discussed above present promising results for the use of sonoporation for tissue regeneration, the lack of current clinical trials may allude to the existence of additional hurdles that need to be addressed in pre-clinical, regeneration-focused research. Despite this, the clinical trials currently utilizing sonoporation show that it is a safe practice in humans, which should lend support to the development of clinical protocols specifically targeting tissue regeneration.

In addition to the limitations addressed above, there are also concerns regarding the scalability of sonoporation, as most of the pre-clinical experiments only utilized small animal models. A recent study on acousticfluidic sonoporation appears to present encouraging results for increasing the scalability of intracellular delivery, achieving a throughput of 200,000 cells per minute in a single channel [24], although large animal studies will be needed to further evaluate this technique. 

A greater understanding of how sonoporation impacts other biochemical processes may be necessary prior to clinical implementation. For example, it has been suggested that more research is needed to characterize the effect that sonoporation has on extracellular vesicles (EVs), as the release of EVs seems to be part of the action to reseal the disrupted plasma membrane, with one study reporting a significant increase in EV release between 2 and 4 h after sonoporation [83].

A few studies have proposed that combining sonoporation with other transfection strategies may have the potential to increase transfection efficiency and the therapeutic benefits of sonoporation. The coordinated use of sonoporation with other gene delivery systems, including viral vector injection [84] or magneto-sonoporation [85], may further enhance the regenerative potential of sonoporation [81], as suggested in a recent study [70]. It is evident that sonoporation has the potential to play a large role in the future of regenerative medicine but will benefit from a greater foundation of pre-clinical research. 

## Figures and Tables

**Table 1:** Advantages and disadvantages of non-viral gene delivery methods.

Delivery Method	Mechanism	Advantages	Limitations	References
Naked DNA Injection	Direct injection of DNA at targeted site	Simplest and least expensive delivery method, localized DNA uptake	Poor and variable expression levels, damage to tissue surrounding injection site	[19]
Gene-Activated Matrix	Scaffolds implanted for extended release of DNA at targeted site	Directed and sustained gene expression, both in vivo and ex vivo approaches available, 3D template for tissue regeneration	May require other viral or non-viral vectors to increase expression, possible DNA damage during scaffold formation	[20]
Magnetofection	Magnetic particles complexed with DNA and an external magnetic field	Fast delivery of nucleic acids, high transduction efficiency, low-dose requirements	Localization can be difficult in vivo, particle size impacts cell entry, cytotoxicity	[6]
Electroporation	High voltage electric pulses to increase membrane permeability	High throughput, low cost, more efficient than naked DNA injection or sonoporation	Variable transfection efficiency, limited cell viability, non-homogenous tissue regeneration, potential tissue damage	[21,22]
Sonoporation	Ultrasound waves create pores in cell membrane due to cavitation	Noninvasive, less tissue damage compared to electroporation, ultrasound is highly accepted in the clinical setting, more efficient than naked DNA injection, systemic injection is possible	Low transfection efficiency, cell membrane damage is possible, low reproducibility	[23]

**Table 2:** The use of sonoporation for tissue regeneration.

Regeneration Model	Animal Model	References
Bone Regeneration	Mouse	[22,31,35,43]
	Pig	[39]
Soft Tissue-Bone Integration	Pig	[40]
Myocardial Angiogenesis	Mouse	[44,45]
	Rat	[46,47]
Peripheral Angiogenesis	Rabbit	[48]
	Rat	[49,50,51,52]
Pancreatic Islet Regeneration	Rat	[53,54,55,56]

**Table 3:** The use of sonoporation for skeletal tissue regeneration.

Model	Animal	Ultrasound	Frequency (MHz)	Conclusion	References
Ectopic	Mouse	Rich-Mar Sonitron 2000	1	Sonoporation applied with intramuscular injection of rhBMP-9 plasmid and lipid-stabilized microbubbles resulted in ectopic bone formation	[22]
		Rich-Mar Sonitron 2000		Repeated sonoporation with BMP-2 plasmid significantly increased osteoinduction compared to one treatment session	[43]
		Sonidel SP100		Using 4 W/cm^2^ sonoporation and constitutive BMP2/7 co-expression plasmid significantly increased ectopic bone formation, but with variable morphology and irregular shape	[35]
		Sonidel SP100		Use of a GAM and BMP2/7 co-expression plasmid significantly enhanced ectopic bone formation compared to standard sonoporation	[31]
Femur Defect	Rat	Sonidel SP100	1	Use of a BMP2/7 co-expression plasmid resulted in fracture union in 33% of rats, compared to the 0% union rate in the control group, although this result was not statistically significant	[35]
Tibia Defect	Pig	Philips Sonos 5500; S3 transducer	1.3	Using a collagen scaffold and hBMP-6 plasmid led to complete radiographic and functional healing, similar to that shown with autograft implantation	[39]
ACL Reconstruction	Pig	Philips Sonos 5500; S3 transducer	1.3	Collagen scaffold and BMP-6 plasmid injection significantly enhanced osteointegration and tissue continuity, with no ectopic bone formation	[40]

**Table 4:** The use of sonoporation for cardiac tissue regeneration.

Model	Animal	Ultrasound	Frequency (MHz)	Conclusion	References
Ischemia/reperfusion (I/R) Injury	Mouse	Siemens Acuson Sequoia C256; 15L8 transducer	8	Injection of either VEGF or SCF plasmids resulted in greater capillary and arteriolar density, myocardial perfusion, and enhanced cardiac function compared to the control group	[44]
		Philips Sonos 5500; S12 transducer	7	Myocardial perfusion and ventricular function improved progressively with the number of treatments of stem cell factor (SCF) and stromal cell-derived factor-1α (SDF-1α) plasmids	[45]
	Rat	Siemens Acuson Sequoia C256; 15L8 transducer	8	Cationic microbubble delivery of the AKT gene produced the greatest increase in ventricular function and myocardial perfusion, resulting in decreased infarct size and reducing apoptosis	[46]
		GE Healthcare Vivid 7; M3S transducer	1.6	Antagomir delivery to the myocardium is dependent on ultrasound frequency and mode, and delivery primarily occurred at the anterior wall of the heart	[47]

**Table 5:** The use of sonoporation for ischemia in peripheral tissue.

Model	Animal	Ultrasound	Frequency (MHz)	Conclusion	References
Hindlimb ischemia	Rabbit	Not specified	1	Angiographic score and capillary density of animals treated with ultrasound and HGF plasmid was significantly greater than the control, resulting in a significant increase in blood flow and blood pressure ratio	[48]
	Rat	Philips Sonos 5500; S3 transducer	1.3	Infusion of VEGF-165 plasmid resulted in significant improvement in microvascular blood flow and increased vessel density, with transfection localized predominantly to the vascular endothelium of arterioles	[49]
				Both IM and IV delivery of VEGF-165 plasmid produced significant increases in microvascular blood volume and blood flow, but microvascular blood flow was greater in IV-treated animals	[50]
				Temporally separated VEGF and Ang-1 plasmid delivery resulted in increased blood flow, vessel density, and sustained an increase in flow reserve	[51]
				Treatment with miR-126-3p resulted in significant improvements in microvascular perfusion, and repeated treatment exhibited an even greater angiogenic response	[52]

**Table 6:** The use of sonoporation as a therapy for diabetes.

Model	Animal	Ultrasound	Frequency (MHz)	Conclusion	References
STZ-induced diabetes	Rat	Philips Sonos 5500; S3 transducer	1.3	RIP3.1-NeuroD1 plasmid promoted islet regeneration from surviving beta-cells, with normalization of glucose, insulin, and C-peptide levels up to 30 days, but pretreating with SP600125 could extend the duration of islet regeneration and normoglycemia to 90 days	[53]
				Injection of the Nkx2.2 gene induced robust proliferation and differentiation of adult pancreatic progenitors, curing STZ-induced diabetes for 3 months	[54]
				A single sonoporation treatment with cyclin D2/CDK4/GLP-1 plasmids induced β-cell regeneration with reversal of diabetes for 6 months without evidence of toxicity or activation of oncogenes	[55]
				ANGPTL8 gene targeted to the pancreas significantly alleviated but did not totally reverse STZ-induced diabetes, but treatment did promote the proliferation of adult and aged beta cells, expanding the beta-cell mass and improving glucose tolerance	[56]

## References

[1] Jacques E., Suuronen E.J. (2020). The Progression of Regenerative Medicine and its Impact on Therapy Translation. Clin. Transl. Sci..

[2] Jessop Z.M., Al-Sabah A., Francis W.R., Whitaker I.S. (2016). Transforming healthcare through regenerative medicine. BMC Med..

[3] Fang Y.L., Chen X.G., Godbey W.T. (2015). Gene delivery in tissue engineering and regenerative medicine. J. Biomed. Mater. Res. Part B Appl. Biomater..

[4] Ruiz M.M., Regueiro J.R. (2012). New Tools in Regenerative Medicine: Gene Therapy. Adv. Exp. Med. Biol..

[5] Bleiziffer O., Eriksson E., Yao F., Horch R.E., Kneser U. (2007). Gene transfer strategies in tissue engineering. J. Cell. Mol. Med..

[6] Sung Y.K., Kim S.W. (2019). Recent advances in the development of gene delivery systems. Biomater. Res..

[7] Waehler R., Russell S.J., Curiel D.T. (2007). Engineering targeted viral vectors for gene therapy. Nat. Rev. Genet..

[8] Bouard D., Alazard-Dany N., Cosset F.-L. (2009). Viral vectors: From virology to transgene expression. Br. J. Pharmacol..

[9] Liu Y., Wang D.-A. (2014). Viral vector-mediated transgenic cell therapy in regenerative medicine: Safety of the process. Expert Opin. Biol. Ther..

[10] Venkatesan J.K., Rey-Rico A., Cucchiarini M. (2019). Current Trends in Viral Gene Therapy for Human Orthopaedic Regenerative Medicine. Tissue Eng. Regen. Med..

[11] Alaee F., Sugiyama O., Virk M.S., Tang H., Drissi H., Lichtler A.C., Lieberman J.R. (2013). Suicide gene approach using a dual-expression lentiviral vector to enhance the safety of ex vivo gene therapy for bone repair. Gene Ther..

[12] Nayerossadat N., Ali P.A., Maedeh T. (2012). Viral and nonviral delivery systems for gene delivery. Adv. Biomed. Res..

[13] Wolff J.A., Budker V. (2005). The Mechanism of Naked DNA Uptake and Expression. Adv. Genet..

[14] Schillinger U., Wexel G., Hacker C., Kullmer M., Koch C., Gerg M., Vogt S., Ueblacker P., Tischer T., Hensler D. (2008). A Fibrin Glue Composition as Carrier for Nucleic Acid Vectors. Pharm. Res..

[15] Kaipel M., Schützenberger S., Hofmann A.T., Ferguson J., Nau T., Redl H., Feichtinger G.A. (2014). Evaluation of fibrin-based gene-activated matrices for BMP2/7 plasmid codelivery in a rat nonunion model. Int. Orthop..

[16] Plank C., Schillinger U., Scherer F., Bergemann C., Remy J.-S., Krötz F., Anton M., Lausier J., Rosenecker J. (2003). The Magnetofection Method: Using Magnetic Force to Enhance Gene Delivery. Biol. Chem..

[17] Ramamoorth M., Narvekar A. (2015). Non viral vectors in gene therapy—An overview. J. Clin. Diagn. Res..

[18] Zu H., Gao D. (2021). Non-viral Vectors in Gene Therapy: Recent Development, Challenges, and Prospects. AAPS J..

[19] Lu Q.L., Liang H.-D., Partridge T., Blomley M.J.K. (2003). Microbubble ultrasound improves the efficiency of gene transduction in skeletal muscle in vivo with reduced tissue damage. Gene Ther..

[20] D’Mello S., Atluri K., Geary S.M., Hong L., Elangovan S., Salem A.K. (2017). Bone Regeneration Using Gene-Activated Matrices. AAPS J..

[21] Gantenbein B., Tang S., Guerrero J., Higuita-Castro N., Salazar-Puerta A.I., Croft A.S., Gazdhar A., Purmessur D. (2020). Non-viral Gene Delivery Methods for Bone and Joints. Front. Bioeng. Biotechnol..

[22] Sheyn D., Kimelman-Bleich N., Pelled G., Zilberman Y., Gazit D., Gazit Z. (2007). Ultrasound-based nonviral gene delivery induces bone formation in vivo. Gene Ther..

[23] Tomizawa M., Shinozaki F., Motoyoshi Y., Sugiyama T., Yamamoto S., Sueishi M. (2013). Sonoporation: Gene transfer using ultrasound. World J. Methodol..

[24] Belling J.N., Heidenreich L.K., Tian Z., Mendoza A.M., Chiou T.-T., Gong Y., Chen N.Y., Young T.D., Wattanatorn N., Park J.H. (2020). Acoustofluidic sonoporation for gene delivery to human hematopoietic stem and progenitor cells. Proc. Natl. Acad. Sci. USA.

[25] Rychak J.J., Klibanov A.L. (2014). Nucleic acid delivery with microbubbles and ultrasound. Adv. Drug Deliv. Rev..

[26] Fechheimer M., Boylan J.F., Parker S., Sisken J.E., Patel G.L., Zimmer S.G. (1987). Transfection of mammalian cells with plasmid DNA by scrape loading and sonication loading. Proc. Natl. Acad. Sci. USA.

[27] Tachibana K., Uchida T., Ogawa K., Yamashita N., Tamura K. (1999). Induction of cell-membrane porosity by ultrasound. Lancet.

[28] Klibanov A.L. (2006). Microbubble contrast agents: Targeted ultrasound imaging and ultrasound-assisted drug-delivery applications. Investig. Radiol..

[29] Muskula P.R., Main M.L. (2017). Safety with Echocardiographic Contrast Agents. Circ. Cardiovasc. Imaging.

[30] Kaur H., Uludag H., El-Bialy T. (2014). Effect of Nonviral Plasmid Delivered Basic Fibroblast Growth Factor and Low Intensity Pulsed Ultrasound on Mandibular Condylar Growth: A Preliminary Study. BioMed Res. Int..

[31] Nomikou N., Feichtinger G., Saha S., Nuernberger S., Heimel P., Redl H., McHale A. (2018). Ultrasound-responsive gene-activated matrices for osteogenic gene therapy using matrix-assisted sonoporation. J. Tissue Eng. Regen. Med..

[32] Nomikou N., Feichtinger G.A., Redl H., McHale A.P. (2016). Ultrasound-mediated gene transfer (sonoporation) in fibrin-based matrices: Potential for use in tissue regeneration. J. Tissue Eng. Regen. Med..

[33] Lentacker I., De Cock I., Deckers R., De Smedt S., Moonen C. (2014). Understanding ultrasound induced sonoporation: Definitions and underlying mechanisms. Adv. Drug Deliv. Rev..

[34] Qin P., Xu L., Han T., Du L., Yu A.C. (2016). Effect of non-acoustic parameters on heterogeneous sonoporation mediated by single-pulse ultrasound and microbubbles. Ultrason. Sonochem..

[35] Feichtinger G.A., Hofmann A.T., Slezak P., Schützenberger S., Kaipel M., Schwartz E., Neef A., Nomikou N., Nau T., van Griensven M. (2014). Sonoporation Increases Therapeutic Efficacy of Inducible and Constitutive BMP2/7 In Vivo Gene Delivery. Hum. Gene Ther. Methods.

[36] Nomikou N., Tiwari P., Trehan T., Gulati K., McHale A.P. (2012). Studies on neutral, cationic and biotinylated cationic microbubbles in enhancing ultrasound-mediated gene delivery in vitro and in vivo. Acta Biomater..

[37] Li Y.S., Davidson E., Reid C.N., McHale A.P. (2009). Optimising ultrasound-mediated gene transfer (sonoporation) in vitro and prolonged expression of a transgene in vivo: Potential applications for gene therapy of cancer. Cancer Lett..

[38] Nishida K., Doita M., Takada T., Kakutani K.-I., Miyamoto H., Shimomura T., Maeno K., Kurosaka M. (2006). Sustained Transgene Expression in Intervertebral Disc Cells In Vivo Mediated by Microbubble-Enhanced Ultrasound Gene Therapy. Spine.

[39] Bez M., Sheyn D., Tawackoli W., Avalos P., Shapiro G., Giaconi J.C., Da X., Ben David S., Gavrity J., Awad H.A. (2017). In situ bone tissue engineering via ultrasound-mediated gene delivery to endogenous progenitor cells in mini-pigs. Sci. Transl. Med..

[40] Bez M., Kremen T., Tawackoli W., Avalos P., Sheyn D., Shapiro G., Giaconi J.C., Ben David S., Snedeker J., Gazit Z. (2018). Ultrasound-Mediated Gene Delivery Enhances Tendon Allograft Integration in Mini-Pig Ligament Reconstruction. Mol. Ther..

[41] Li Y.S., Reid C.N., McHale A.P. (2008). Enhancing ultrasound-mediated cell membrane permeabilisation (sonoporation) using a high frequency pulse regime and implications for ultrasound-aided cancer chemotherapy. Cancer Lett..

[42] Perez R., Won J.-E., Knowles J.C., Kim H.-W. (2013). Naturally and synthetic smart composite biomaterials for tissue regeneration. Adv. Drug Deliv. Rev..

[43] Osawa K., Okubo Y., Nakao K., Koyama N., Bessho K. (2009). Osteoinduction by microbubble-enhanced transcutaneous sonoporation of human bone morphogenetic protein-2. J. Gene Med..

[44] Fujii H., Sun Z., Li S.-H., Wu J., Fazel S., Weisel R.D., Rakowski H., Lindner J., Li R.-K. (2009). Ultrasound-Targeted Gene Delivery Induces Angiogenesis After a Myocardial Infarction in Mice. JACC Cardiovasc. Imaging.

[45] Kwekkeboom R.F., Sluijter J., van Middelaar B.J., Metz C.H., Brans M.A., Kamp O., Paulus W.J., Musters R.J. (2016). Increased local delivery of antagomir therapeutics to the rodent myocardium using ultrasound and microbubbles. J. Control. Release.

[46] Fujii H., Li S.-H., Wu J., Miyagi Y., Yau T.M., Rakowski H., Egashira K., Guo J., Weisel R.D., Li R.-K. (2011). Repeated and targeted transfer of angiogenic plasmids into the infarcted rat heart via ultrasound targeted microbubble destruction enhances cardiac repair. Eur. Heart J..

[47] Sun L., Huang C.-W., Wu J., Chen K.-J., Li S.-H., Weisel R.D., Rakowski H., Sung H.-W., Li R.-K. (2013). The use of cationic microbubbles to improve ultrasound-targeted gene delivery to the ischemic myocardium. Biomaterials.

[48] Taniyama Y., Tachibana K., Hiraoka K., Aoki M., Yamamoto S., Matsumoto K., Nakamura T., Ogihara T., Kaneda Y., Morishita R. (2002). Development of safe and efficient novel nonviral gene transfer using ultrasound: Enhancement of transfection efficiency of naked plasmid DNA in skeletal muscle. Gene Ther..

[49] Leong-Poi H., Kuliszewski M.A., Lekas M., Sibbald M., Teichert-Kuliszewska K., Klibanov A.L., Stewart D.J., Lindner J.R. (2007). Therapeutic Arteriogenesis by Ultrasound-Mediated VEGF_165_ Plasmid Gene Delivery to Chronically Ischemic Skeletal Muscle. Circ. Res..

[50] Kobulnik J., Kuliszewski M.A., Stewart D.J., Lindner J.R., Leong-Poi H. (2009). Comparison of Gene Delivery Techniques for Therapeutic Angiogenesis: Ultrasound-Mediated Destruction of Carrier Microbubbles Versus Direct Intramuscular Injection. J. Am. Coll. Cardiol..

[51] Smith A.H., Kuliszewski M.A., Liao C., Rudenko D., Stewart D.J., Leong-Poi H. (2012). Sustained Improvement in Perfusion and Flow Reserve After Temporally Separated Delivery of Vascular Endothelial Growth Factor and Angiopoietin-1 Plasmid Deoxyribonucleic Acid. J. Am. Coll. Cardiol..

[52] Cao W.J., Rosenblat J.D., Roth N.C., Kuliszewski M.A., Matkar P.N., Rudenko D., Liao C., Lee P.J., Leong-Poi H. (2015). Therapeutic Angiogenesis by Ultrasound-Mediated MicroRNA-126-3p Delivery. Arter. Thromb. Vasc. Biol..

[53] Chen S., Shimoda M., Wang M.-Y., Ding J., Noguchi H., Matsumoto S., Grayburn P.A. (2010). Regeneration of pancreatic islets in vivo by ultrasound-targeted gene therapy. Gene Ther..

[54] Chen S., Shimoda M., Chen J., Matsumoto S., Grayburn P.A. (2012). Ectopic transgenic expression of NKX2.2 induces differentiation of adult pancreatic progenitors and mediates islet regeneration. Cell Cycle.

[55] Chen S., Shimoda M., Chen J., Matsumoto S., Grayburn P.A. (2012). Transient overexpression of cyclin D2/CDK4/GLP1 genes induces proliferation and differentiation of adult pancreatic progenitors and mediates islet regeneration. Cell Cycle.

[56] Chen J., Chen S., Huang P., Meng X.-L., Clayton S., Shen J.-S., Grayburn P.A. (2015). In vivo targeted delivery of ANGPTL8 gene for beta cell regeneration in rats. Diabetologia.

[57] Henkel J., Woodruff M., Epari D., Steck R., Glatt V., Dickinson I.C., Choong P., Schuetz M.A., Hutmacher D.W. (2013). Bone Regeneration Based on Tissue Engineering Conceptions—A 21st Century Perspective. Bone Res..

[58] Kimelman-Bleich N., Pelled G., Zilberman Y., Kallai I., Mizrahi O., Tawackoli W., Gazit Z., Gazit D. (2011). Targeted Gene-and-host Progenitor Cell Therapy for Nonunion Bone Fracture Repair. Mol. Ther..

[59] Aihara H., Miyazaki J.-I. (1998). Gene transfer into muscle by electroporation in vivo. Nat. Biotechnol..

[60] Nakashima M., Mizunuma K., Murakami T., Akamine A. (2002). Induction of dental pulp stem cell differentiation into odontoblasts by electroporation-mediated gene delivery of growth/differentiation factor 11 (Gdf11). Gene Ther..

[61] Liao Z.-K., Tsai K.-C., Wang H.-T., Tseng S.-H., Deng W.-P., Chen W.-S., Hwang L.-H. (2011). Sonoporation-mediated anti-angiogenic gene transfer into muscle effectively regresses distant orthotopic tumors. Cancer Gene Ther..

[62] McMahon J.M., Wells K.E., Bamfo J.E., Cartwright M.A., Wells D.J. (1998). Inflammatory responses following direct injection of plasmid DNA into skeletal muscle. Gene Ther..

[63] Betz O.B., Betz V.M., Nazarian A., Egermann M., Gerstenfeld L.C., Einhorn T., Vrahas M.S., Bouxsein M.L., Evans C.H. (2007). Delayed administration of adenoviral BMP-2 vector improves the formation of bone in osseous defects. Gene Ther..

[64] Delalande A., Bureau M.-F., Midoux P., Bouakaz A., Pichon C. (2010). Ultrasound-assisted microbubbles gene transfer in tendons for gene therapy. Ultrasonics.

[65] Shapiro G., Wong A.W., Bez M., Yang F., Tam S., Even L., Sheyn D., Ben-David S., Tawackoli W., Pelled G. (2016). Multiparameter evaluation of in vivo gene delivery using ultrasound-guided, microbubble-enhanced sonoporation. J. Control. Release.

[66] Frantz S., Bauersachs J., Ertl G. (2008). Post-infarct remodelling: Contribution of wound healing and inflammation. Cardiovasc. Res..

[67] Chen S., Shimoda M., Chen J., Grayburn P.A. (2012). Stimulation of adult resident cardiac progenitor cells by durable myocardial expression of thymosin beta 4 with ultrasound-targeted microbubble delivery. Gene Ther..

[68] Kopechek J.A., McTiernan C.F., Chen X., Zhu J., Mburu M., Feroze R., Whitehurst D.A., Lavery L., Cyriac J., Villanueva F.S. (2019). Ultrasound and Microbubble-targeted Delivery of a microRNA Inhibitor to the Heart Suppresses Cardiac Hypertrophy and Preserves Cardiac Function. Theranostics.

[69] Liu Y., Li L., Su Q., Liu T., Ma Z., Yang H. (2015). Ultrasound-Targeted Microbubble Destruction Enhances Gene Expression of microRNA-21 in Swine Heart via Intracoronary Delivery. Echocardiography.

[70] Wang W., Tayier B., Guan L., Yan F., Mu Y. (2022). Pre-transplantation of Bone Marrow Mesenchymal Stem Cells Amplifies the Therapeutic Effect of Ultrasound-Targeted Microbubble Destruction–Mediated Localized Combined Gene Therapy in Post-Myocardial Infarction Heart Failure Rats. Ultrasound Med. Biol..

[71] Bastarrachea R.A., Chen J., Kent J.W., Nava-Gonzalez E.J., Rodriguez-Ayala E., Daadi M.M., Jorge B., Laviada-Molina H., Comuzzie A.G., Chen S. (2017). Engineering brown fat into skeletal muscle using ultrasound-targeted microbubble destruction gene delivery in obese Zucker rats: Proof of concept design. IUBMB Life.

[72] Nakashima M., Tachibana K., Iohara K., Ito M., Ishikawa M., Akamine A. (2003). Induction of Reparative Dentin Formation by Ultrasound-Mediated Gene Delivery of Growth/Differentiation Factor 11. Hum. Gene Ther..

[73] Nakashima M. (1994). Induction of Dentin Formation on Canine Amputated Pulp by Recombinant Human Bone Morphogenetic Proteins (BMP)-2 and -4. J. Dent. Res..

[74] Sugano M., Negishi Y., Endo-Takahashi Y., Hamano N., Usui M., Suzuki R., Maruyama K., Aramaki Y., Yamamoto M. (2013). Gene delivery to periodontal tissue using Bubble liposomes and ultrasound. J. Periodontal Res..

[75] Liao A.-H., Huang Y.-J., Chuang H.-C., Wang C.-H., Shih C.-P., Chiang C.-P. (2021). Minoxidil-Coated Lysozyme-Shelled Microbubbes Combined with Ultrasound for the Enhancement of Hair Follicle Growth: Efficacy In Vitro and In Vivo. Front. Pharmacol..

[76] Ryu J.-Y., Won E.-J., Lee H.A.R., Kim J.H., Hui E., Kim H.P., Yoon T.-J. (2020). Ultrasound-activated particles as CRISPR/Cas9 delivery system for androgenic alopecia therapy. Biomaterials.

[77] Jiang Z.-Z., Xia G.-Y., Zhang Y., Dong L., He B.-Z., Sun J.-G. (2013). Attenuation of hepatic fibrosis through ultrasound-microbubble-mediated HGF gene transfer in rats. Clin. Imaging.

[78] Yang D., Gao Y.-H., Tan K.-B., Zuo Z.-X., Yang W.-X., Hua X., Li P.-J., Zhang Y., Wang G. (2013). Inhibition of hepatic fibrosis with artificial microRNA using ultrasound and cationic liposome-bearing microbubbles. Gene Ther..

[79] Snipstad S., Vikedal K., Maardalen M., Kurbatskaya A., Sulheim E., Davies C.D.L. (2021). Ultrasound and microbubbles to beat barriers in tumors: Improving delivery of nanomedicine. Adv. Drug Deliv. Rev..

[80] Idbaih A., Canney M., Belin L., Desseaux C., Vignot A., Bouchoux G., Asquier N., Law-Ye B., Leclercq D., Bissery A. (2019). Safety and Feasibility of Repeated and Transient Blood-Brain Barrier Disruption by Pulsed Ultrasound in Patients with Recurrent Glioblastoma. Clin. Cancer Res..

[81] Yang L., Yan F., Ma J., Zhang J., Liu L., Guan L., Zheng H., Li T.-S., Liang D., Mu Y. (2019). Ultrasound-Targeted Microbubble Destruction-Mediated Co-Delivery of Cxcl12 (Sdf-1alpha) and Bmp2 Genes for Myocardial Repair. J. Biomed. Nanotechnol..

[82] Be P.P., Alam M., Li S., Chow S.K.H., Zheng Y. (2021). Low-Intensity Pulsed Ultrasound Stimulation for Bone Fractures Healing: A Review. J. Ultrasound Med..

[83] Yuana Y., Jiang L., Lammertink B.H.A., Vader P., Deckers R., Bos C., Schiffelers R.M., Moonen C.T. (2017). Microbubbles-Assisted Ultrasound Triggers the Release of Extracellular Vesicles. Int. J. Mol. Sci..

[84] Bazan-Peregrino M., Rifai B., Carlisle R.C., Choi J., Arvanitis C.D., Seymour L.W., Coussios C.C. (2013). Cavitation-enhanced delivery of a replicating oncolytic adenovirus to tumors using focused ultrasound. J. Control. Release.

[85] Filippi M., Dasen B., Scherberich A. (2021). Rapid Magneto-Sonoporation of Adipose-Derived Cells. Materials.

